# Evaluating the Prevalence and Incidence of Bronchiectasis and Nontuberculous Mycobacteria in South Korea Using the Nationwide Population Data

**DOI:** 10.3390/ijerph18179029

**Published:** 2021-08-27

**Authors:** Da-In Park, Sungchan Kang, Seunghye Choi

**Affiliations:** 1 Department of Nursing, College of Life Science and Nano Technology, Hannam University, Daejeon 34430, Korea; dpark@hnu.kr; 2 Graduate School of Public Health, Seoul National University, Seoul 08826, Korea; rjmcmc@gmail.com; 3 College of Nursing, Gachon University, Incheon 21936, Korea

**Keywords:** bronchiectasis, nontuberculous mycobacteria, prevalence, incidence, population study

## Abstract

Although the prevalence and incidence of bronchiectasis are rising worldwide, basic epidemiologic data have not been reported in Korea. Therefore, this study was conducted to investigate epidemiological characteristics of bronchiectasis and NTM (nontuberculous mycobacteria) pulmonary diseases in Korea using the National Health Insurance Service-National Sample Cohort (NHIS-NSC) data. The relative risks of prevalence and incidence after adjusting for demographic characteristics were evaluated by multivariate Poisson regression. The result of this study showed the prevalence and incidence rates of bronchiectasis and NTM to be epidemiologically similar to each other with a few slight differences, while the prevalence rate of bronchiectasis was not significantly different by gender, and its incidence rate was significantly lower in women than in men. Both the prevalence and incidence of NTM were significantly higher in women than in men. Both the prevalence and incidence rates of bronchiectasis and NTM were significantly lower in the age group below 40–49 years, and significantly higher in the age groups thereafter. As there were gender differences of bronchiectasis and NTM, gender-sensitive risk management should be available. In addition, since both bronchiectasis and NTM increase in prevalence and incidence after the age of 40–49, early detection and intervention strategies targeting the appropriate age group are needed.

## 1. Introduction

Bronchiectasis, a chronic progressive and irreversible respiratory condition of diverse etiology, is characterized by structurally abnormal bronchial dilation, persistent airway infection, and recurrent exacerbations [1]. Its prevalence continues to increase around the globe, and is placing a significant burden on individuals and health care systems. The clinical symptoms of bronchiectasis include chronic cough, sputum production, hemoptysis, and recurrent pulmonary exacerbations, and ineffective management of the condition may lead to airway obstruction and breathing difficulties [2]. Although symptoms may vary depending on the severity of bronchial dilation, those who are affected have difficulties excreting secretions from the lower airways, which further leads to increased chance of inflammation and infection caused by bacterial overgrowth.

The lower respiratory tract normally maintains its sterile condition, but those with bronchiectasis are easily and chronically colonized by microorganisms such as yeasts, fungi, and nontuberculous mycobacteria (NTM) [3]. The NTM is a type of bacteria that is commonly found in the natural environment and has potential to cause infections in respiratory systems, lymph nodes, skin and soft tissues, bones, etc. The most commonly affected is the respiratory system, accounting for more than 90% of all NTM infections [4,5]. The criteria for the diagnosis of NTM lung disease include both clinical symptoms (i.e., pulmonary symptoms, nodular or cavitary opacities on chest radiograph, or a high-resolution computed tomography scan that shows multifocal bronchiectasis with multiple small nodules and appropriate exclusion of other diagnosis) and microbiologic evidence (i.e., positive culture results, etc.) [5]. The incidence of NTM lung disease is increasing worldwide in association with an increase in chronic lung disease patients and an increase in immunocompromised patients after chemotherapy or organ transplantation [6].

Once the patient develops NTM pulmonary infection, the airways are further damaged and dilated, causing a vicious cycle of worsening bronchiectasis. A previous study has reported that a history of respiratory infection with NTM increases the chance of bronchiectasis development, especially among underweight women above middle age [7]. The incidence of NTM infection in patient with bronchiectasis is continuously rising, but limited data are available on the prevalence and factors associated with NTM in bronchiectasis. Due to its chronicity and recurrent exacerbations, the quality of life impairment in these patients is reported to be equivalent to those with severe chronic obstructive pulmonary disease [8].

It has been reported that there has been an annual increase of 8.7% in the prevalence of bronchiectasis in the U.S., with a significantly higher prevalence among Asian-Americans [9]. Although the prevalence rates varied within different countries, studies have also suggested an increasing pattern in the European region [1,10]. Overall, diagnosis of the respiratory condition is reported to be significantly higher among older people, females, and Asians [1,11]. Gender has been identified as a strong risk factor where females are more likely to present symptoms of bronchiectasis in an earlier phase, while males do not present symptoms until older age [12]. A previous cohort study conducted in Taiwan also indicated the prevalence of bronchiectasis to be dominant in women [13]. Additionally, a Singaporean cohort study identified the preponderance of women [14]. However, contradicting results have also been identified in some studies, suggesting further investigation. Despite study results indicating Asians to be at higher risk, there is a lack of comprehensive prevalence datasets and extensive research in the Asia-Pacific region [10]. A previous study conducted in Korea reported that 9.1% of patients undergoing medical examinations to have bronchiectasis [14]. Similar to reports from the U.S. and European countries, bronchiectasis in Korea has also indicated its relations to age, gender, a history of tuberculosis, and accompanying respiratory diseases [10,15]. Although this study investigated the prevalence and associated factors of bronchiectasis, it only analyzed patient data collected at one singular medical center, which limits generalizability to the whole Korean population. Furthermore, despite the importance of bronchiectasis treatment, only a few drugs have been approved for use [11], and interest in this disease is relatively low in Korea. Furthermore, basic epidemiological data and even standard treatment guidelines have not been developed yet.

As mentioned earlier, NTM pulmonary disease is highly likely to further develop into bronchiectasis. Thus, it is necessary to congruently examine these two conditions as well as to identify factors that are associated with the conditions. Hence, the purpose of this study was to investigate the prevalence and incidence rate of bronchiectasis and NTM pulmonary diseases and associated factors in Korea using the sample cohort data from the National Health Insurance Service-National Sample Cohort (NHIS-NSC) data.

## 2. Materials and Methods

### 2.1. Procedure

This observational retrospective cohort study was conducted using the National Health Insurance Service-NHIS-2020-2-030 database (version 2.0). Since the Korean health insurance system is mandatory for all citizens by law, all data related to the use of health care systems and pharmacies are stored and managed by the National Health Insurance Service [16]. The National Health Insurance Corporation sample cohort contains socio-demographic and clinical information of 1,025,340 participants, representing 2.2% of the total population, and systematically stratified and randomized sampling of domestic patients from 2002 to 2015 ensure representativeness [17].

#### 2.1.1. Research Subject Selection

Data from the National Health Insurance Service sample cohort (NHIS-NSC 2002–2015) were used to refer to all claim data, except for non-covered treatment performed in Korea during 2007–2015. Since the frequency of bronchiectasis and NTM was tracked from 2007, we analyzed the 2007–2015 data, and tuberculosis medication users were counted from 2002. The diagnosis of NTM and bronchiectasis was defined based on the Korean Standard Classification of Causes of Disease (KCD-8), which was modified from the International Classification of Diseases (ICD-10) [18].

In order to investigate the trends in the incidence of bronchiectasis and NTM by year, only newly diagnosed patients were considered. We extracted those who were newly diagnosed with bronchiectasis or NTM and received outpatient treatment or hospitalized from January 2009 and survived. We excluded those who were hospitalized or who received outpatient treatment for bronchiectasis or NTM during the two years between 2007 and 2008.

#### 2.1.2. Criteria for Exclusion of Study Subjects

The study subjects were excluded according to the criteria of EMBARC (European Multicenter Audit and Research Collaboration), a European bronchiectasis research network. Patients with bronchiectasis due to cystic fibrosis, diffuse interstitial lung disease, lung resection, and heart and lung transplantation were excluded [12].

### 2.2. Samples

#### Epidemiological Characteristics Investigation Methods

**Figure 1:**
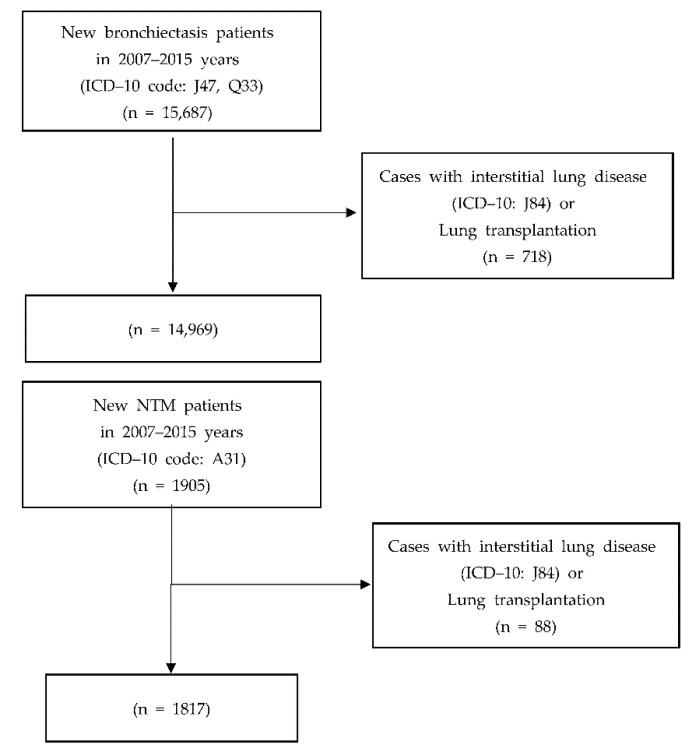
Flowchart for the study population selection.

Data were classified into bronchiectasis if diagnosed at least once with the main disease J47 (bronchiectasis) or Q33 (congenital malformations of lung). NTM was classified when the main disease was diagnosed as A31.0 (NTM) at least once. Finally, tuberculosis was classified if the drug efficacy classification code 622 was present in the treatment history (30 table) or if the prescription issuance details (60 table) showed as a susceptible disease. Additionally, cases with interstitial lung disease (J84) in the main or sub-disease or Q8101/Q8102 (single/double lung transplantation) in the treatment code were excluded (Figure 1).

From 2007 to 2015, the actual number of patients who met the diagnostic criteria were counted, and the prevalence was calculated for all people in the year according to gender, age groups, regions, and income levels.

Age groups were divided with 10-year intervals, starting from 20–29 group up to the 80 and older group. The prevalence and incidence rates per 100,000 populations for each age group were obtained by dividing the number of bronchiectasis patients in the year for each age group by the total population of the sample cohort and multiplying by 100,000 [19]. Classification by region was divided into a total of 10 regions, which includes the capital city and nine provinces (Seoul, Gangwon-do, Gwangju-Jeollanam-do, Daegu-Gyeongsangbuk-do, Busan-Ulsan-Gyeongsangnam-do, Incheon-Gyeonggi-do, Jeollabuk-do, Jeju-do, Chungnam-Daejeon-Sejong, and Chungcheongbuk). The incidence rate by region was investigated, and the income level was classified into five levels based on the deciles of insurance premiums, with 0–20th being the lowest income [19].

### 2.3. Ethical Considerations

The National Health Insurance Service sample cohort (NHIS-NSC 2002–2013) database (version 2.0) is a standardized dataset for policy and academic research purpose, and any personal identifier information was deleted prior to researchers’ access to the dataset so that the subjects could not be identified. In the case of using data related to the public use of health care systems and pharmacies that are legally stored and managed by the National Health Insurance Corporation under the health insurance system, consent for research cannot be obtained, and thus written consent was exempted. The study’s retrospective protocol was approved by the institutional review board of Gachon University (1044396-201910-HR-180-01).

### 2.4. Statistical Analysis

The relative risks of prevalence and incidence after adjusting for demographic characteristics, regions, and income levels were evaluated by multivariate Poisson regression. We initially performed univariable analysis for the response variable and explanatory variables (sex, age group, income levels, residence area, etc.). Then, we fitted the multivariable model. Poisson regression was used to model the number of events in the population. In the model for prevalence, we coded the annual number of patients in each subgroup population, which was stratified by explanatory variables as the dependent variable and we added a natural logarithm of population number at risk as offset. In the case of the model for incidence, the number of new patients in each year were coded as dependent variable and natural logarithm of person-year value were used as the offset variable. All statistical analyses were performed using the SAS software version 9.4 (SAS Institute Inc., Cary, NC, USA). Statistical significance was set at *p* < 0.05.

## 3. Results

### 3.1. Bronchiectasis Prevalence/Incidence

**Figure 2:**
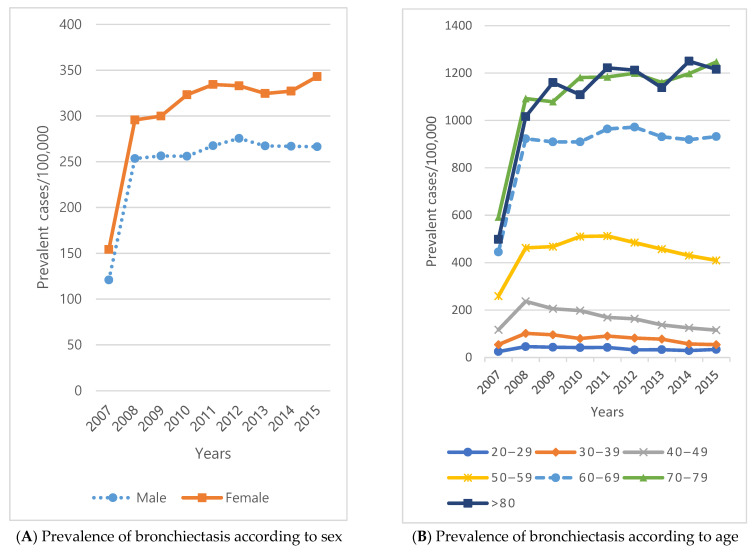

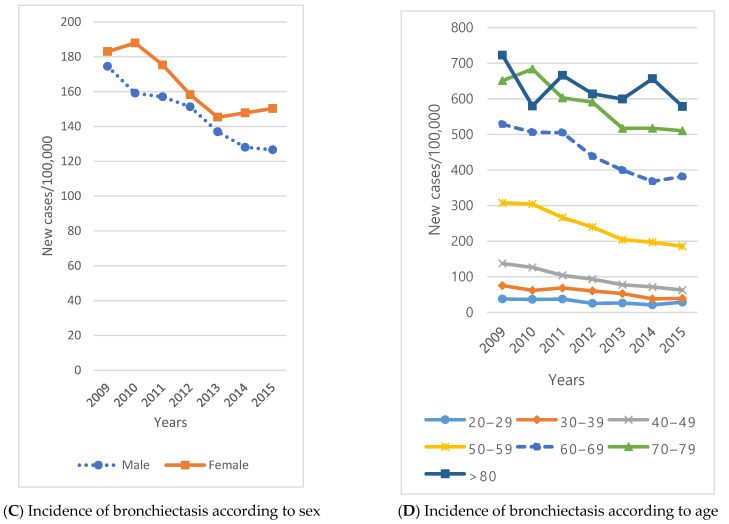
Temporal trends of bronchiectasis prevalence and incidence by sex and age.

In the sample cohort, from 2007 to 2015, the prevalence of bronchiectasis increased from 120.9 to 266.4 per 100,000 males and from 154.3 to 343.1 for females (Figure 2A). The incidence rate decreased from 174.5 to 126.6 in males from 2009 to 2015 and decreased from 183 to 150.3 in females (Figure 2C). However, the incidence rate was higher in females (Figure 2C). The regional prevalence rate was the highest in Gangwon-do.

### 3.2. NTM Prevalence/Incidence

**Figure 3:**
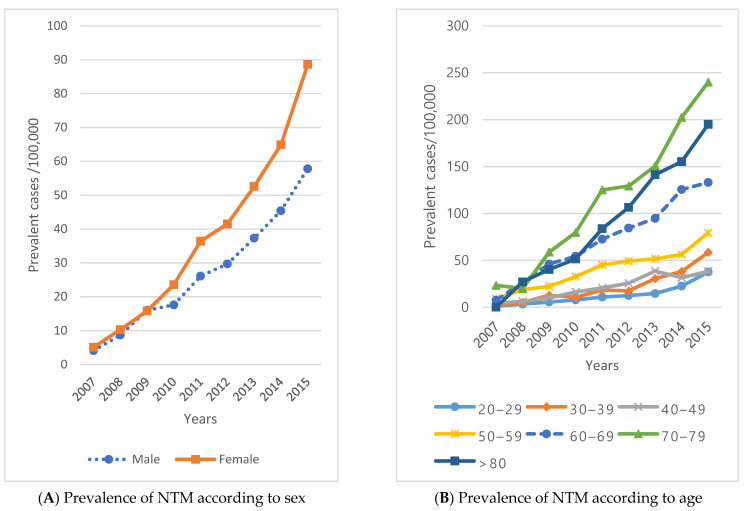

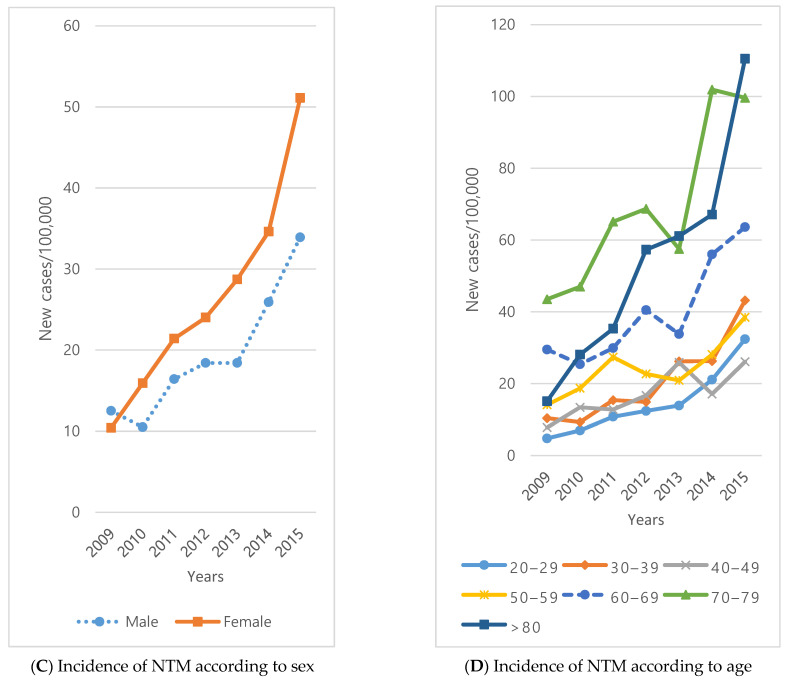
Temporal trends of NTM prevalence and incidence by sex and age.

The prevalence of NTM increased from 4.1 to 57.8 per 100,000 males and from 5.1 to 88.6 for females (Figure 3A). The incidence rate increased from 12.5 to 33.9 in males from 2009 to 2015 and increased from 10.4 to 51.1 in females (Figure 3C). The incidence rate was higher in males, but the incidence rate in females showed a gradual increasing pattern (Figure 3C).

### 3.3. Relative Risk of Bronchiectasis

The relative risk of bronchiectasis prevalence was not significantly different by gender, and the incidence rate was significantly lower in women than in men. Both the prevalence and incidence rates of bronchiectasis were significantly lower in the age groups younger than 40–49 years, and significantly higher in the age groups thereafter. When looking at regional differences, the prevalence and incidence rates of bronchiectasis were higher in non-metropolitan regions compared to the capital city, Seoul. The prevalence was higher when the income decile was higher than the 7th decile (with the lowest decile being 1). The incidence rate was higher in the 8th and 10th deciles than in the 1st decile. Both the prevalence and incidence rates were higher among workers under occupational insurance compared to those under local insurance (Table 1).

### 3.4. Relative Risk of NTM

Both the prevalence and incidence rates of NTM were significantly higher in women than in men. Both the prevalence and incidence rates of NTM were significantly lower in the age groups below the ages of 40–49, and significantly higher in the age groups thereafter. However, there was no significant difference between the age ranges of 30–39 and 40–49. When looking at regional differences, the prevalence of NTM was lower in non-metropolitan areas. However, the NTM incidence rate was lower in only a few regions (Gangwon/Gwangju, Jeonnam/Busan, Ulsan, and Gyeongnam/Jeonbuk). The prevalence was higher in the 5th, 8th, 9th, and 10th deciles (with the lowest decile being 1). The incidence was higher in the 8th, 9th, and 10th quartiles than in the 1st quartile. Both the prevalence and incidence rates were higher among workers under occupational insurance compared to those under local insurance (Table 2).

## 4. Discussion

Our study indicates that the prevalence, incidence, and relative risks of bronchiectasis and NTM varied depending on several demographic factors. Similar to previous epidemiological studies conducted in the U.S. and European countries, our study also showed gender differences. Although a decreasing pattern in prevalence was identified in both genders within the six-year period, the incidence rate in women remained higher than that of men throughout. As for the NTM, although males had a higher incidence rate, female incidence rate gradually increased. The sexual dichotomy in bronchiectasis is reported to be multifactorial. Although our study results showed higher incidence of NTM in males, the clinical outcomes and disease symptoms may have been more severe in females. Studies have indicated that in non-cystic fibrosis bronchiectasis, females are more likely to have more severe respiratory complications, poorer prognosis, and worse lung functions compared to males [20,21]. The gender differences observed in bronchiectasis patients are partly due to physical and biological differences. Females tend to have smaller lungs and conducting bronchial airways. Given that pseudostratified ciliated epithelial tissue with mucus-secreting properties is located in the conducting airways, females have less secretion of mucus, which serves as the first line of defense against inhaled pathogens such as NTM [20,22]. Lower incidence in women in our study may also be associated with gender bias in the diagnosis of bronchiectasis. Studies have indicated that females tend to have delayed diagnosis, which further delays initiation of appropriate treatment [20,23].

Both the prevalence and incidence rates for bronchiectasis and NTM were significantly lower in age groups under 40–49 and significantly higher in age groups after that. Our results correspond with other studies that indicated older age to be a risk factor of bronchiectasis and NTM [1,7,10]. Similar to our results, a study conducted on a multicenter cohort in Spain also identified age ≥ 50 to be independently associated with NTM [24]. The relationship between older age, bronchiectasis, and NTM is highly likely to be prolonged, repeated exposure to environmental factors that negatively affect the respiratory condition. Additionally, impaired pulmonary function due to natural aging may be another contributing factor. The socio-economical characteristics also took part as a contributing factor. The prevalence and incidence rates of bronchiectasis were significantly higher in areas other than Seoul, the capital city. On the other hand, the NTM was significantly less prevalent in a few regions other than Seoul. Similar differences based on the location of residence was observed in a previous population-based cohort study in the UK [25].

The prevalence and incidence rates due to socioeconomic differences were similar in bronchiectasis and NTM. In terms of income levels, the prevalence of bronchiectasis was higher in the 7th deciles and higher (with the lowest decile being 1), and the prevalence of NTM was higher in the 5th, 8th, 9th, and 10th deciles (with the lowest decile being 1). The incidence of bronchiectasis was higher in the 8th and 10th quartiles than in the 1st quartile, and the NTM incidence was higher in the 8th, 9th, and 10th quartiles than in the 1st quartile. Both the prevalence and incidence rates of bronchiectasis and NTM were higher among workers under occupational insurance compared to those under local insurance. Bronchiectasis or NTM is known as a chronic disease that causes economic burden [26]. The economic burden of bronchiectasis may not be just pharmaceutical, hospitalization, and treatment costs, but also include indirect and intangible costs associated with ongoing symptoms and exacerbations on patient health related quality of life and well-being. Thus, the actual economic burden may be larger than what has been previously reported [27]. As a result of this study, the prevalence and incidence rates of bronchiectasis or NTM were higher in the group of relatively high-income deciles, but greater awareness of health management of these diseases is required.

The main strength of this study is that we analyzed the NHIS-NSC data, which provides satisfactory representativeness of the Korean population and data accuracy [17]. Additionally, to our knowledge, this is the first study to comprehensively evaluate the bronchiectasis and NTM in Korea using longitudinal population-based national cohort data. However, a few limitations should be taken into consideration. First, the severity of the respiratory conditions was not considered, which limits our interpretation of gender differences. Second, clinical information related to bronchiectasis and NTM such as smoking habits, onset, duration, extent, respiratory function, or microbiologic test results were not available for data analysis. Therefore, we were limited to perfume analyses adjusted for known confounding variables. Finally, we only included those who survived after first diagnosis and treatment. Therefore, those with severe condition or serious complications may have been lost in our analysis. Despite the limitations above-mentioned, our results provide population-based data on bronchiectasis and NTM and emphasize the importance of appropriate and urgent treatment of this patient group.

## 5. Conclusions

This study evaluated the prevalence and incidence rate and associated factors of bronchiectasis and NTM in Korea using a national sample cohort dataset. As the incidence and prevalence rates of bronchiectasis and NTM continue to increase with the national and global aging phenomenon, further research and investment in identifying patient-centered treatment plan is greatly needed. Despite Asians having a greater risk factor, bronchiectasis and NTM are somewhat neglected pulmonary conditions in the Asian region. In order to decrease economic burden and to increase quality of life, further cohort studies should be conducted to provide better understanding of etiologies, microbiology, and treatments for bronchiectasis and NTM.

## Figures and Tables

**Table 1:** Poisson regression to estimate the relative risk of annual prevalence and incidence rate of bronchiectasis.

	Prevalence Rate				Incidence Rate			
	RR	95% CI		*p*-Value	RR	95% CI		*p*-Value
		Lower Limit	Upper Limit			Lower Limit	Upper Limit	
Female	0.99	0.97	1.00	0.116	0.95	0.92	0.98	0.004
Male (ref)	1.00				1			
Age group								
0–9	0.12	0.11	0.13	<0.001	0.13	0.11	0.15	<0.001
10–19	0.17	0.16	0.18	<0.001	0.16	0.14	0.18	<0.001
20–29	0.26	0.25	0.27	<0.001	0.28	0.25	0.31	<0.001
30–39	0.55	0.53	0.57	<0.001	0.54	0.5	0.59	<0.001
50–59	2.66	2.59	2.72	<0.001	2.56	2.41	2.72	<0.001
60–69	4.66	4.53	4.81	<0.001	4.55	4.29	4.82	<0.001
70–79	6.78	6.62	6.96	<0.001	6.2	5.83	6.59	<0.001
>80	8.10	7.85	8.41	<0.001	7.23	6.7	7.8	<0.001
40–49 (ref)	1				1			
Residence								
(1)	2.05	1.97	2.12	<0.001	2.04	1.88	2.20	<0.001
(2)	1.20	1.16	1.23	<0.001	1.21	1.13	1.30	<0.001
(3)	1.15	1.12	1.19	<0.001	1.16	1.09	1.24	<0.001
(4)	1.07	1.04	1.09	<0.001	1.15	1.09	1.22	<0.001
(5)	1.02	0.99	1.04	0.203	1.01	0.96	1.06	0.770
(6)	1.25	1.21	1.30	<0.001	1.28	1.17	1.39	<0.001
(7)	1.14	1.06	1.22	0.001	1.21	1.03	1.41	0.018
(8)	1.07	1.03	1.11	<0.001	1.1	1.02	1.19	0.010
(9)	1.27	1.22	1.32	<0.001	1.33	1.21	1.46	<0.001
(10) (ref)	1				1			
Income, decile								
2nd	1.02	0.98	1.06	0.324	0.99	0.90	1.08	0.776
3rd	0.99	0.95	1.03	0.677	1.03	0.94	1.12	0.564
4th	0.97	0.93	1.01	0.147	0.95	0.87	1.03	0.233
5th	1.01	0.97	1.05	0.627	1	0.92	1.09	0.979
6th	0.99	0.96	1.03	0.694	1.04	0.96	1.13	0.350
7th	1.04	1.01	1.08	0.021	1.04	0.96	1.13	0.309
8th	1.10	1.06	1.14	<0.001	1.12	1.04	1.21	0.002
9th	1.04	1.01	1.07	0.013	1.06	0.98	1.13	0.147
10th	1.14	1.11	1.19	<0.001	1.1	1.02	1.18	0.011
1st (ref)	1				1			
Types of insurance								
(A)	1.24	1.22	1.26	<0.001	1.17	1.13	1.21	<0.001
(B) (ref)	1				1			

CI, Confidence interval; (1), Gangwon-do; (2), Gwangju-Jeollanam-do; (3), Daegu-Gyeongsanbuk-do; (4), Busan-Ulsan-Gyeongsangnam-do; (5), Incheon-Gyeonggi-do; (6), Jeollabuk-do; (7), Jeju-do; (8), Chungnam-Daejeon-Sejong; (9), Chungcheongbuk-do; (10), Seoul; (A), Employee health insurance; (B), Self-employed health insurance.

**Table 2:** Poisson regression to estimate the relative risk of annual prevalence and incidence rate of NTM.

	Prevalence Rate				Incidence Rate			
	RR	95% CI		*p*-Value	RR	95% CI		*p*-Value
		Lower Limit	Upper Limit			Lower Limit	Upper Limit	
Female	1.24	1.19	1.31	<0.001	1.26	1.15	1.39	<0.001
Male (ref)	1				1			
Age group								
0–9	0.77	0.68	0.88	<0.001	0.82	0.66	1.03	0.087
10–19	0.33	0.28	0.38	<0.001	0.33	0.25	0.44	<0.001
20–29	0.61	0.54	0.69	<0.001	0.77	0.62	0.95	0.013
30–39	1.08	0.98	1.2	0.120	1.05	0.88	1.26	0.575
50–59	1.95	1.77	2.14	<0.001	1.7	1.43	2.03	<0.001
60–69	2.85	2.59	3.16	<0.001	2.54	2.13	3.03	<0.001
70–79	4.97	4.53	5.47	<0.001	4.61	3.87	5.5	<0.001
>80	4.54	3.97	5.16	<0.001	4.23	3.32	5.37	<0.001
40–49 (ref)	1				1			
Residence								
(1)	0.48	0.39	0.58	<0.001	0.56	0.4	0.78	<0.001
(2)	0.66	0.59	0.74	<0.001	0.62	0.49	0.77	<0.001
(3)	0.85	0.77	0.92	<0.001	1.04	0.89	1.21	0.647
(4)	0.34	0.3	0.38	<0.001	0.36	0.3	0.44	<0.001
(5)	1.05	0.98	1.13	0.158	0.97	0.85	1.1	0.618
(6)	0.61	0.53	0.72	<0.001	0.62	0.47	0.83	0.001
(7)	0.55	0.41	0.75	<0.001	0.62	0.37	1.04	0.070
(8)	0.67	0.59	0.75	<0.001	0.83	0.68	1.01	0.062
(9)	0.6	0.5	0.71	<0.001	0.51	0.36	0.72	0.001
(10) (ref)	1				1			
Income, decile								
2nd	1.1	0.95	1.27	0.195	1.27	1	1.63	0.054
3rd	1.13	0.98	1.3	0.094	1.04	0.8	1.35	0.797
4th	0.92	0.79	1.06	0.262	0.87	0.66	1.13	0.298
5th	1.18	1.03	1.35	0.016	1.11	0.87	1.42	0.391
6th	1.12	0.98	1.27	0.104	1.13	0.89	1.43	0.316
7th	1.11	0.97	1.26	0.123	1.08	0.85	1.36	0.526
8th	1.22	1.07	1.38	0.002	1.28	1.03	1.6	0.027
9th	1.34	1.19	1.51	<0.001	1.34	1.09	1.67	0.007
10th	1.47	1.31	1.65	<0.001	1.25	1.01	1.55	0.038
1st (ref)	1				1			
Types of insurance								
(A)	1.59	1.49	1.68	<0.001	1.54	1.39	1.71	<0.001
(B) (ref)	1							

CI, Confidence interval; (1), Gangwon-do; (2), Gwangju-Jeollanam-do; (3), Daegu-Gyeongsanbuk-do; (4), Busan-Ulsan-Gyeongsangnam-do; (5), Incheon-Gyeonggi-do; (6), Jeollabuk-do; (7), Jeju-do; (8), Chungnam-Daejeon-Sejong; (9), Chungcheongbuk-do; (10), Seoul; (A), Employee health insurance; (B), Self-employed health insurance.

## Data Availability

The NHIS-NSC database access on http://nhiss.nhis.or.kr/bd/ab/bdaba021eng.do (accessed on 22 October 2020–20 March 2021) requires a completed application form, a research proposal, and the institutional review board’s approval document.
